# Ab initio phasing macromolecular structures using electron-counted MicroED data

**DOI:** 10.1038/s41592-022-01485-4

**Published:** 2022-05-30

**Authors:** Michael W. Martynowycz, Max T. B. Clabbers, Johan Hattne, Tamir Gonen

**Affiliations:** 1 Howard Hughes Medical Institute, University of California, Los Angeles, CA USA; 2 Department of Biological Chemistry, University of California, Los Angeles, CA USA; 3 Department of Physiology, University of California, Los Angeles, CA USA

**Keywords:** Proteins, Cryoelectron microscopy, Molecular biophysics

## Abstract

Structures of two globular proteins were determined ab initio using microcrystal electron diffraction (MicroED) data that were collected on a direct electron detector in counting mode. Microcrystals were identified using a scanning electron microscope (SEM) and thinned with a focused ion beam (FIB) to produce crystalline lamellae of ideal thickness. Continuous-rotation data were collected using an ultra-low exposure rate to enable electron counting in diffraction. For the first sample, triclinic lysozyme extending to a resolution of 0.87 Å, an ideal helical fragment of only three alanine residues provided initial phases. These phases were improved using density modification, allowing the entire atomic structure to be built automatically. A similar approach was successful on a second macromolecular sample, proteinase K, which is much larger and diffracted to a resolution of 1.5 Å. These results demonstrate that macromolecules can be determined to sub-ångström resolution by MicroED and that ab initio phasing can be successfully applied to counting data.

## Abstract (web summary)

This article reports sub- and near-atomic structures of triclinic lysozyme and serine protease proteinase K, respectively, providing first demonstrations of ab initio phasing using electron counted MicroED data to solve macromolecular structures.

## Main

The cryogenic electron microscopy method MicroED can be used to determine the atomic structures of inorganic materials, small organic molecules, natural products, soluble proteins and membrane proteins from vanishingly small crystals^1^. The data are collected as a movie on a fast camera while the stage of the electron microscope is continuously rotating the crystal in a parallel electron beam^2^. As the method is analogous to the rotation method in X-ray crystallography^3^, data processing is conducted using standard X-ray crystallography software. For MicroED of small molecules and short peptides, which typically diffract to atomic resolution, initial phases are commonly determined using ab initio direct methods^4^ or from radiation-induced damage in special circumstances^5^. Phases for electron diffraction can also be determined from images recorded on a transmission electron microscope (TEM), as demonstrated for two-dimensional membrane protein crystals^6–8^. For MicroED data from three-dimensional macromolecular crystals, phases have thus far only been determined by molecular replacement^1^. Molecular replacement for MicroED data has been demonstrated with distant homologs^9,10^ or fragments extracted from homologs^11^, but there have been no reports of successful phasing in the absence of a previously known model. A major obstacle for phasing using idealized fragments in macromolecular MicroED data has been phase improvement. To date, density-modification algorithms have persistently failed to improve the maps for macromolecular MicroED (for example, ref. ^11^). Thus, even if idealized fragments had been placed accurately, phasing was intractable. Robust phasing by any means other than molecular replacement has remained elusive for macromolecular MicroED^1^.

MicroED data have been collected using a variety of camera systems and microscopes. While charge-coupled devices can be used^12^, these cameras tend to be slow and relatively insensitive, which makes low-dose data collection by continuous rotation difficult. Cameras based on complementary metal oxide semiconductor (CMOS) technology allow faster readout and often have better signal-to-noise ratios^1,2,13^. The shorter dead time between frames allows CMOS cameras to operate in rolling-shutter mode, which facilitates continuous-rotation experiments^2^. Hybrid pixel detectors have also been successful for collecting macromolecular MicroED data^10,14^. Hybrid pixel detectors have smaller arrays of large pixels that can complicate data collection when working with large unit cells such as proteins. By contrast, direct electron detectors offer distinct advantages and were pivotal for the ‘resolution revolution’ in single-particle cryogenic electron microscopy^15,16^. In electron-counting mode, these low-noise cameras can detect individual electrons. The increased accuracy of electron counting in combination with faster readout rates promises to deliver superior data quality for MicroED. Direct electron detectors have been used in prior MicroED investigations but only in integrating (linear) mode^17,18^.

**Fig. 1:**
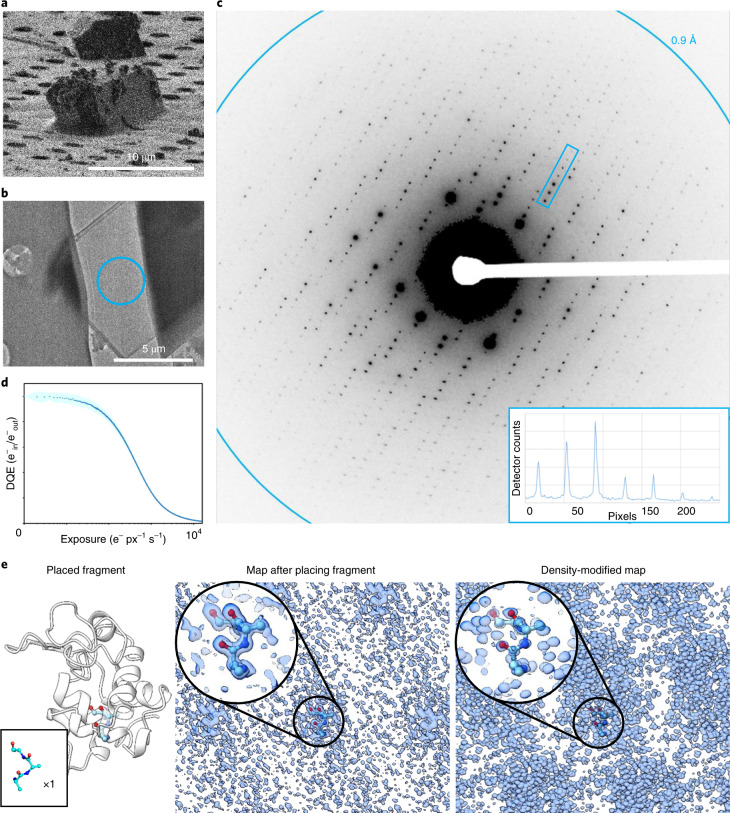
Electron-counted MicroED data from milled lamellae of lysozyme. **a**, A typical lysozyme microcrystal imaged using the FIB. **b**, A thin, milled lamella from **a** identified with the TEM. **c**, MicroED data collected in counting mode using a direct electron detector extending to atomic resolution. The data were summed over a two-degree wedge for display purposes only. A line plot through the box indicated in **c** is inset, demonstrating the quality of the counting mode data collected showing high peak intensity over background, *I*/*σ*_*Ι*_. **d**, Simulated detector quantum efficiency (DQE) (e^−^_in_/e^−^_out_) for this camera operating in counting mode. px, pixel. **e**, The ab initio phasing strategy in which a small fragment was placed and the initial phases were extended by density modification. The position of the placed fragment and the maps before and after density modification are displayed as indicated to the right. Source data

Here, a structure of triclinic lysozyme at 0.87 Å using a Falcon 4 direct electron detector (Thermo Fisher Scientific) in electron-counting mode is reported (Fig. 1). The exposure rate, frame rate and total exposure time of the MicroED experiment were optimized to allow data collection within the specifications of the camera (Methods and Supplementary Fig. 1). Using this approach, far superior data were collected when compared with previous studies, and phases were determined ab initio using an idealized fragment of only 15 atoms. Initial phases from these atoms were improved by density modification^19^. A model was built automatically into this map using standard crystallographic software^20^ without user intervention. To test the robustness of this approach, data were collected from crystals of a serine protease, proteinase K (Supplementary Fig. 2). The data extended to a resolution of 1.5 Å with better statistics than those previously reported for structures of this protein using other detectors^21,22^. This structure was initially phased by automatically placing four ideal helices followed by successive rounds of density modification and backbone tracing. The backbone model was then completed by automated means. This study demonstrates electron-counting MicroED data collection to sub-ångström resolution using a direct electron detector. Even at near-atomic resolutions, density modification could be successfully applied, leading to ab initio phasing of macromolecular MicroED data. These structures set new benchmarks for the achievable quality of MicroED data and increase the scope of possible experiments targeting proteins with unknown structures.

## Results

### Preparing lysozyme crystals for MicroED experiments

Crystals of triclinic lysozyme were grown in batch (Methods). The crystals were visible under a light microscope and were initially about 10 µm in size. A slurry of these microcrystals was vitrified on electron microscopy grids. Grids were loaded into a dual-beam FIB–SEM where individual crystals that were at least 5 µm from a grid bar and at least three grid squares away from the edge of the grid were identified. The crystals were then coated in multiple layers of platinum for protection from the ion beam during the milling process. Each crystal was then milled into a thin lamella approximately 300 nm in thickness, about the ideal thickness for MicroED data collection at an accelerating voltage of 300 kV based on prior mean free path measurements^23^ (Fig. 1a,b and Methods).

The milled lamellae were then loaded under cryogenic conditions into a Titan Krios TEM. Lamellae were identified by low-magnification montages in which each site appeared as a semi-transparent shape suspended above an empty strip. Eighteen of the 20 milled lamellae were found in the TEM; two did not survive the cryotransfer step. For each lamella, the eucentric height was carefully adjusted. Lamellae were inspected initially at an intermediate magnification to verify that the sample was intact and that no contamination had built up (Fig. 1b). Each lamella was checked for diffraction, and the camera length was adjusted depending on the attainable resolution before data collection.

### Collecting MicroED data in counting mode

MicroED data were collected in electron-counting mode using a Falcon 4 direct electron detector (Methods and Supplementary Fig. 1c,d). This camera allows for highly accurate detection of single electron events. However, the number of electrons that can be counted in each pixel of each frame is limited. To ensure accurate reporting of the intensities, the exposure rate must be kept very low. This strategy reduces errors caused by too many electrons hitting the same pixel within a readout cycle of the detector but risks missing weak reflections in the background. These stringent requirements were met by greatly reducing the exposure rate and compensating by increasing the total exposure time (Methods). This strategy prevents the strong reflections from overwhelming or damaging the detector while weak high-resolution reflections are sampled at sufficient frequency to recover their intensities (Fig. 1d and Supplementary Fig. 1).

Multiple settings had to be adjusted to achieve a suitably low exposure rate for these experiments. Importantly, the camera’s dose protector, which automatically retracts the camera when the microscope enters diffraction mode, must be disabled (Methods). The smallest second condenser lens aperture (C2) was coupled with the highest spot size possible on our instrument (50 µm and spot size 11). The instrument was kept in microprobe mode to avoid an approximately fivefold increase in exposure rate that occurs by deactivating the condenser mini lens. Because these experiments were conducted on a Titan Krios, the beam size could be changed while maintaining a near-perfect parallel illumination. The beam diameter was spread to 25 µm to further reduce the exposure per unit area. Together, these modifications reduced the total exposure per area by a factor of up to 10× compared to prior experiments on this instrument^24^. Further, the data were collected as a 420-s exposure at 250 frames per second, which is currently the longest duration allowed by the detector software. Combined with a very low rotation rate of the stage at either 0.15° or 0.2° per second, data collection covered a total real space wedge of approximately 60° or 80°, respectively (Fig. 1c). In this manner, the exposure was spread in space and time, allowing accurate measurements of single electron events even in diffraction mode. As a result, these datasets had total exposures up to 4× lower than prior investigations for similar macromolecules even though the recording time here was more than twice as long^25^. The total exposure per lamella was ~0.64 e^−^ Å^−2^. The background noise and total flux on the camera were further reduced by using a selected area aperture of 100 µm that corresponds to a region of about 2 µm in diameter on the specimen. Under these conditions, essentially all pixel values fall within the linear range of the detector and are all well below the threshold for damaging the detector (Methods).

### Solving lysozyme at subatomic resolution

The movies from the Falcon 4 were sliced into either 1.0-s or 0.5-s segments, 420 or 840 individual frames, each 2,048 × 2,048 16-bit pixels in size, spanning a rotation of 0.075–0.2°. Images were converted to SMV format using the MicroED tools^13^ adapted for the Falcon 4–EPU-D–Velox metadata format. The total size of a compressed MicroED movie in counting mode for these exposures is typically 2.2 GiB (up to 14,455 frames during 420 s). The converted frames were processed using standard crystallographic software^26,27^. The space group for all 18 lysozyme MicroED datasets was found to be *P*1, and the unit cell was determined to be (*a*, *b*, *c*) = (26.42 ± 0.15 Å, 30.72 ± 0.30 Å, 33.01 ± 0.21 Å), (*α*, *β*, *γ*) = (88.32 ± 0.25°, 109.09 ± 0.38°, 112.07 ± 0.32°) (Table 1). The data were merged to increase completeness. The subsequent merging steps identified two lysozyme lamellae that correlated poorly with the other 16 integrated datasets. These two datasets were discarded. A high-resolution cutoff at 0.87 Å was applied, corresponding to the point where the CC_1/2_, the correlation coefficient between randomly chosen half-datasets^28^, was still significant at the 0.1% level. The overall completeness of the final dataset was 87.5%, with all resolution shells below the resolution of 1 Å being >95% complete (Table 1, Supplementary Table 1 and Supplementary Fig. 3).

**Table 1:** MicroED crystallographic table of triclinic lysozyme

MicroED structure of triclinic lysozyme	
	EMD 25184
	PDB 7SKW
Accelerating voltage (kV)	300
Electron exposure (e^–^ Å^−2^)	0.64
Wavelength (Å)	0.0197
No. crystals	16
Resolution range (Å)	16.05–0.87 (0.9011–0.87)
Space group	*P*1
Unit cell (*a*, *b*, *c*) (Å)	26.42 ± 0.15, 30.72 ± 0.30, 33.01 ± 0.21
(*α*, *β*, *γ*) (°)	88.32 ± 0.25, 109.09 ± 0.38, 112.07 ± 0.32
Total reflections (no.)	569,407 (5,797)
Unique reflections (no.)	64,986 (2,783)
Multiplicity	8.8 (2.1)
Completeness (%)	87.55 (37.64)
*I*/*σ*_*I*_	6.23 (0.66)
Wilson *B* factor	9.44
*R*_merge_	0.2363 (1.035)
*R*_meas_	0.248 (1.409)
*R*_pim_	0.0730 (0.9451)
CC_1/2_	0.99 (0.147)
CC*	0.998 (0.506)
Reflections used in refinement (no.)	64,955 (2,783)
Reflections used for *R*_free_ (no.)	3,165 (128)
*R*_work_	0.1969
*R*_free_	0.2214
No. non-hydrogen atoms	1,190
Macromolecules	1,018
Ligands	16
Solvent	156
Protein residues (no.)	129
r.m.s._bonds_	0.027
r.m.s._angles_	2.2
Ramachandran favored (%)	98.43
Ramachandran allowed (%)	1.57
Ramachandran outliers (%)	0
Rotamer outliers (%)	0.93
Clashscore	5.44
Average *B* factor	14.39
Macromolecules	10.93
Ligands	16.51
Solvent	36.77

EMD, Electron Microscopy Data Bank; PDB, Protein Data Bank. Values in parentheses in column 2 denote the highest resolution shell.

**Fig. 2:**
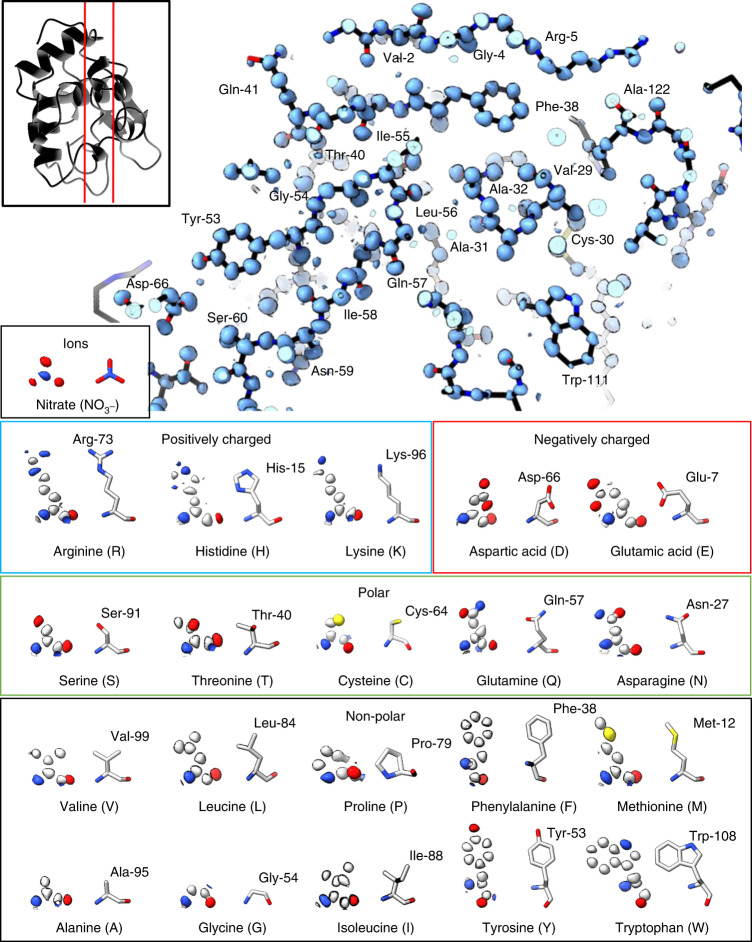
Ab initio structure of triclinic lysozyme at a resolution of 0.87 Å. Top, a slice through the final structure of triclinic lysozyme as black sticks with the density-modified map using normalized structure factors shown in blue. The location of the slice through the final structure is indicated in the inset on the top left. Bottom, examples of 20 amino acids and NO_3_^−^ from the final structure are displayed with their normalized structure factor map at a resolution of 0.87 Å from density modification. The maps are contoured between the 1*σ* and 2*σ* level for each individual amino acid carved to 1 Å for best visibility of the non-hydrogen atoms.

Phasing was performed by automatically placing an idealized helical triple-alanine fragment followed by density modification (Fig. 1e and Methods). A single helical fragment of only three alanine residues was sufficient to determine the entire lysozyme structure. After automatic placement in Phaser^29^, an interpretable map was produced following 144 rounds of dynamic density modification^19^, resulting in a map showing individually resolved atoms throughout the entire unit cell where all residues and several NO_3_^−^ ions could be unambiguously identified (Fig. 2 and Supplementary Videos 1 and 2). The density-modified map (*E*, *φ*) was similar to the final (2*mF*_o_ − *DF*_c_, *φ*) map after refinement. A complete model was automatically built into the density-modified map given only the sequence^20^ and without consulting their known structures (Fig. 2). For this structure, two C-terminal residues and several solvent-exposed side chains were either partially or entirely absent in the map, even after final refinement. They were also poorly resolved in X-ray investigations of triclinic lysozyme at similar resolutions^30^. This structure of lysozyme is overall similar to other structures of triclinic lysozyme determined by X-ray crystallography^30^ (Supplementary Fig. 4). The final model was refined^31^ using electron scattering factors.

### Determining proteinase K structure at near-atomic atomic resolution

The results from lysozyme using electron-counted MicroED data are very promising. However, it is not entirely surprising that sub-ångström data could be determined ab initio even considering the larger size of lysozyme compared to that of small molecules or peptides. To test if other electron-counted MicroED data would perform similarly, this approach was tested again using a sample of the serine protease proteinase K, which is much larger and more representative of the average size of globular proteins in the cell. The crystals were grown in batch, and sample preparation was very similar to the approach used for the triclinic lysozyme microcrystals. The crystals were identically milled and screened (Supplementary Fig. 2). Four proteinase K crystals were found on a single grid that met the selection criteria. MicroED data were collected with an overall similar approach using electron counting (Methods). We similarly found that essentially all pixel values fell within the linear range of the detector and were well below the threshold for damaging the detector (Supplementary Fig. 1 and Methods).

The data were indexed, integrated and scaled using the same software as that used for the triclinic lysozyme data. The Laue class was determined to be 4/*mmm* with a unit cell of (*a* = *b*, *c*) = (67.08 ± 0.21 Å, 106.78 ± 0.36 Å), (*α* = *β* = *γ*) = (90 ± 0°). Two of the four proteinase K lamellae integrated with lower internal consistency and were therefore discarded. A high-resolution cutoff was applied using the same criteria at 1.5 Å, and the overall completeness of the final dataset was 98.8% (Supplementary Tables 2 and 3 and Supplementary Fig. 5). The overall statistics for these merged crystals were better than those from any prior investigations on the same microscope using a CMOS detector^23^.

Phasing larger proteins below atomic resolution is more challenging. The same approach was used as that for lysozyme for phasing, namely, automatically placing idealized helices and then improving the solution using density modification. Four 14-residue-long ideal alanine fragments were automatically placed using Phaser^29^. This solution was extended using multiple rounds of chain tracing and density modification^32^, similar to the procedure implemented by ARCIMBOLDO_LITE^32,33^. The correct space group was determined by attempting initial fragment placing in all possible space groups for this point group and then tracing the best solutions. The solution in space group *P*4_3_2_1_2 yielded a model in which nearly the entire protein backbone was successfully built. In the final round, this procedure traced 253 of 278 alanine residues (Supplementary Table 2 and Supplementary Fig. 6) and the map showed clear side chain densities. The model was completed automatically^20^ from the backbone trace given only the sequence. The structure was similarly refined^31^ using electron scattering factors.

## Discussion

Ab initio phasing using electron-counted data was successful for both lysozyme and proteinase K following a similar approach. The lysozyme structure was determined from an idealized alanine helical fragment of only 15 atoms, corresponding to less than 1.5% of the structure. The maps calculated from only this small fragment showed density only around the placed atoms and uninterpretable noise elsewhere (Fig. 1e): not even the correct side chains for the three placed residues could be reasonably modeled at this stage. However, the entire protein structure along with solvent ions and water molecules were individually resolved after density modification (Fig. 2). The proteinase K structure was determined using four idealized helices, each made of fourteen alanine residues or 280 atoms (Methods and Supplementary Fig. 6), corresponding to about 13% of the structure. This solution also required density modification after placing the initial fragments. Its lower resolution and larger size meant that this structure required multiple rounds of chain tracing and density modification but was also determined ab initio using electron-counting data. This procedure has previously been attempted several times on non-counting datasets without success. The improved data quality from the electron-counted data was critical to ab initio phasing macromolecular crystals using MicroED data.

This study demonstrates that MicroED data can be collected using a Falcon 4 direct electron detector in counting mode. Previous attempts at collecting MicroED data using counting on a Falcon 3 direct electron detector failed because the Falcon 3 detector is slower (40 Hz) and has a much smaller linear range of only about 1 electron per pixel per second^17^. Therefore, the Falcon 3 was previously used in integrating (or linear) mode in which it operates similarly to other CMOS-based detectors. In the current study, the Falcon 4 direct electron detector was used, which is capable of recording images at 250 Hz. Combining the faster frame rate with an ultra-low-dose spread over a long exposure meant that single electron events could be counted and the recorded intensities could be accurately integrated.

The data presented here were collected from crystal lamellae that were milled to an ideal thickness to match the mean free path of electrons accelerated in 300 kV ^31^. Milling crystals can either be used to make large crystals smaller^12,22,34,35^ or to remove material that embeds small crystals and prevents access for MicroED investigation, such as membrane proteins grown in viscous bicelles^24^ or lipidic cubic phase^36^. Regardless of the reason, milling crystals into an ideal thickness is a good practice and is recommended for extracting the most accurate data^23^. The lysozyme crystals milled in the current study diffracted to sub-ångström resolutions even after being milled by a gallium ion beam. This suggests that milling crystals may preserve the diffractive power of the sample. Indeed, the viability of collecting sub-ångström data from crystalline lamellae further solidifies ion beam milling as the preferred approach for preparing macromolecular crystals for MicroED experiments, as the crystals and surrounding material can be shaped and thinned to ideal thicknesses for any accelerating voltage^23^. Importantly, at a mere 300 nm, these lamellae matched the inelastic mean free path of a 300-keV electron. This reduces the contributions of inelastic scattering and multiple scattering events, likely contributing to greater accuracy in the data.

The results presented here open the door for future investigations of phasing macromolecular protein crystals at or beyond near-atomic resolutions using MicroED data. Attempting to apply direct methods as implemented in, for example, Shake-and-Bake^37,38^ or SHELX^39,40^ is an exciting avenue for future investigations because not all proteins will have helices to place for the starting phases. Further developments in ab initio phasing of MicroED data could be used to both automate the process and extend the necessary resolution to cover more challenging structures, such as membrane proteins that do not routinely reach resolutions better than about 3 Å. Regardless, the ability to probe membrane protein structure by MicroED is advantageous as electrons can probe the charge properties in the sample^41^. The quality of MicroED data obtained by counting would likely be further improved with the addition of an energy filter as this made large differences in the quality previously obtained on integrating detectors^18,41–43^, and this detector is compatible with an energy filter from the same manufacturer^44^. Given the importance of phase improvement in traditional X-ray crystallography^45^, the successful application of density-modification algorithms compatible with MicroED data will be of critical importance for solving structures without known homologs or very difficult structures at lower resolutions.

## Methods

### Materials

Hen egg white lysozyme (*Gallus gallus*) and proteinase K (*Engyodontium album*) were purchased from Sigma-Aldrich and used without further purification. Sodium acetate, pH 4.5, MES–NaOH, pH 6.5, calcium chloride and sodium nitrate stock solutions were used directly from Hampton crystallization kits and diluted using Milli-Q water as needed. The magic triangle kit was purchased from Hampton.

### Crystallization of triclinic lysozyme

Crystals were prepared similarly to the protocol originally detailed by Legrand et al.^46^ and then subsequently described by Heijna et al.^47^. Hen egg white lysozyme was dissolved in 0.2 M NaNO_3_, 0.05 M sodium acetate, pH 4.5, to a concentration of 10 mg ml^−1^. A total volume of approximately 0.5 ml was prepared in a cold room and vortexed at the maximum setting for approximately 1 min immediately after mixing. The tube containing this mixture was left at 4 °C overnight. The next morning, the tube was observed to be nearly filled with an opaque, white suspension. The sample was removed from the cold room, sealed with parafilm and left on a benchtop at room temperature (approximately 23 °C) for 1 week. After this time, very large, clear crystals accumulated on the bottom of the tube, and the remainder of the liquid appeared transparent. Small (1-µl) aliquots from the center of the liquid that appeared clear by eye were found to contain a slurry of small, irregularly shaped crystals when viewed under a light microscope.

### Crystallization of proteinase K

Proteinase K was crystallized as described^48^. Protein powder was dissolved at a concentration of 40 mg ml^−1^ in 20 mM MES–NaOH, pH 6.5. Crystals were formed by mixing a 1:1 ratio of protein solution and a precipitant solution composed of 0.5 M NaNO_3_, 0.1 M CaCl_2_ and 0.1 M MES–NaOH, pH 6.5, in a cold room at 4 °C. Microcrystals grew overnight.

### Grid preparation

Quantifoil Cu 200 R 2/2 holey carbon TEM grids were glow discharged for 30 s at 15 mA on the negative setting immediately before use. Grids were loaded into a Leica GP2 vitrification robot. The robot sample chamber was loaded with filter paper and set to 4 °C and 95% humidity for 1 h before use. Protein crystals (3 µl) from the center of either the proteinase K or lysozyme tubes were applied to the carbon side of the glow-discharged grid and allowed to incubate for 30 s. Grids were then gently blotted from the back for 20 s. For lysozyme, the grids were then immediately plunged into super-cooled liquid ethane. For proteinase K, 3 µl 0.25 M I3C, 0.5 M NaNO_3_, 0.1 M CaCl_2_, 0.1 M MES–NaOH, pH 6.5 was added as described^49^. The proteinase K grids were blotted once more from the back for 20 s and then immediately plunged into liquid ethane. Vitrified grids were stored at liquid nitrogen temperature before further experiments.

### Focused ion beam and scanning electron microscopy

The vitrified grids were inserted into autogrid clips at liquid nitrogen temperature. After clipping, the grids were loaded into a cryotransfer shuttle and inserted into a Thermo Fisher Aquilos dual-beam FIB–SEM operating at liquid nitrogen temperatures. The samples were covered with first fine and then rough coats of sputtered platinum immediately after loading. An additional layer of protective platinum was added using the gas-injection system. Gas-injection system platinum coating was conducted in the mapping position at a working distance of 12 mm. In this way, a platinum layer approximately 1 µm thick was slowly deposited over 30 s and continually monitored using the electron beam.

Whole-grid montages of each grid were taken at low magnification using MAPS software (Thermo Fisher). Crystals on the vitrified grids were identified in the FIB–SEM such that each crystal was not within 5 µm of a grid bar, not within three grid squares of the edge and not within 25 µm of another selected crystal (Fig. 1a and Supplementary Fig. 2a). Twenty such crystals across two grids were prepared over 2 d for lysozyme, and five proteinase K crystals on one grid were prepared in 1 d. Identified crystals were brought to the eucentric position and inspected in both the electron and ion beams. Milling was conducted as described previously^13^. Briefly, each crystal was roughly milled to an approximate thickness of 3 µm and an approximate width of 5–10 µm using an ion beam current of 300 pA and the standard rectangular milling patterns. Each crystal was then finely milled to a thickness of approximately 500 nm and a width of approximately 5–10 µm using an ion beam current of 100 pA. Finally, each lamella was polished using an ion beam current of 10 pA to a thickness of approximately 300 nm and a width of 3–8 µm using a cleaning cross-section. This thickness was found to be optimal for experiments at this accelerating voltage^3^. Each of these steps was typically conducted at 1–5-min intervals, pausing to image the lamellae to reduce the influence of sample drift. The sample was imaged in the ion beam using a current of 1.5 pA between milling steps to realign as needed and to assess the quality and thickness of the finished lamella.

### Setting up the Falcon 4 for diffraction experiments

The dose protector that prevents the operation of this detector while in diffraction mode was disabled by a service engineer before experiments. The Falcon 4 direct electron detector internally operates at 250 frames per second, but every 32nd frame is used to reset the detector and does not result in useful data. Owing to bandwidth limitations, the camera furthermore accumulates at least seven raw frames before transmitting their summed image to the controlling computer, which means that the user can obtain no more than ~35 images per second when collecting data in MRC format. For the data presented here, the frames were internally summed to correspond to either 1.0-s or 0.5-s wedges (240 or 119 raw frames; Supplementary Fig. 1).

The damage threshold for this system is described by a deterioration of the DQE rather than the number of events per unit time. Specifically, the DQE for each pixel will decrease by 10% after a total exposure of 1.5 × 10^9^ electrons. Ultimately, the smaller C2 aperture of 50 µm was chosen, with a spot size of 11 and a beam diameter of 20 or 25 µm, corresponding to a total exposure of ~0.64–1.0 e^−^ Å^−2^. Internally, counting mode operates by returning a single count per electron event that is then normalized by the post-counting gain reference. The resulting real pixel values in the MRC file correspond to unit gain and were multiplied by 32 and rounded to the nearest integer during conversion to SMV format. This is reasonably confirmed by the gain values, estimated between 30 and 36 during data processing. To estimate the number of electrons in each pixel in an individual MRC formatted image, we divided by 32 and rounded to the nearest integer. We simulated the pixel DQE in counting mode and mapped the corresponding values to a histogram of all the pixel values that we measured for the highest exposure dataset (Fig. 1d, Supplementary Code and Supplementary Fig. 1). Comparing the pixel values in the data to counts expected for a given exposure, we found that typical values fall within the linear region and that exposures are usually lower than those for single-particle movies (Supplementary Fig. 1). None of our measurements fell below a DQE of 0.6.

### MicroED data collection

Grids containing milled protein crystals were rotated such that the TEM rotation axis was 90° from the FIB–SEM’s milling axis and then loaded into a cryogenically cooled Thermo Fisher Titan Krios 3Gi TEM operating at an accelerating voltage of 300 kV. Low-magnification montages of each grid were collected at a magnification of 64× and used to locate the milled lamellae. Each lamella was brought to its eucentric position before data collection. MicroED data were collected by continuously rotating the stage at a rate of approximately 0.15° s^−1^ or 0.2° s^−1^ for 420 s, covering a total rotation range of approximately 63° or 84°, respectively. This typically spanned the real space wedge corresponding to −31.5° to +31.5° or −42° to +42°. Data were collected using a 50-µm C2 aperture, a spot size of 11 and a beam diameter of 20 or 25 µm. Under these conditions, the total exposure to each crystal per dataset was approximately 1.0 e^−^ Å^−2^ or 0.64 e^−^ Å^−2^, respectively. The exposure rate was confirmed by collecting an identically long exposure using the same beam size and settings in imaging mode in an empty grid square and collecting the movie in counting mode. This was repeated multiple times and averaged to measure the total exposure accurately. Diffraction data were isolated from a small area from the middle of each lamella of approximately 2 µm in diameter at the specimen level using the selected area aperture of 100 µm to remove unwanted background noise. All data were collected using twofold binning and internally summed such that each image recorded either a 1.0-s or 0.5-s exposure spanning approximately 0.075–0.2° of rotation. In this manner, each image stack contained either 420 or 840 images, the last of which was discarded. A single sweep of continuous-rotation MicroED data was collected from each lysozyme lamella. For proteinase K, two sweeps were collected: a high-resolution dataset at a nominal camera distance of 960 mm and then a subsequent low-resolution dataset collected identically but at the longest possible nominal camera distance of 4,300 mm. The post-column magnification on this system is 1.81×. The low-resolution pass was conducted after the high-resolution pass covering a resolution range from approximately 60 to 5 Å.

### MicroED data processing

Movies in MRC format were converted to SMV format using a parallelized version of the MicroED tools^13^ (https://cryoem.ucla.edu/downloads). Each dataset was indexed and integrated using XDS. All datasets were scaled using XSCALE^50^ and xscale_isocluster^51^. Datasets that were of either much poorer resolution or with scaling correlation below 90% were discarded. The uncertainty in unit cell parameters for the merged data was taken to be the standard deviation in the measured unit cells. For both crystals, the space group was verified using POINTLESS^52^. For lysozyme, the data were merged without scaling using AIMLESS^52^, the subsequent intensities were converted to amplitudes in CTRUNCATE^27^, normalized structure factors were calculated using ECALC^27^, and a 5% fraction of the reflections was assigned to a free set using FREERFLAG software packages distributed in the CCP4 program suite^27^.

### Phasing

The lysozyme data could be phased using either Fragon^53^ or Phaser^29^, followed by ACORN^19,54^. An initial phasing solution was achieved using example parameters from the ACORN documentation (http://legacy.ccp4.ac.uk/html/acorn.html#example9). Here, a small fragment of idealized α-helix is used for molecular replacement, and then the best solution is subjected to density modification. In this manner, the number of atoms was systematically lowered in the idealized helix from the initial 50, and we were able to achieve a phasing solution with as few as 15 total atoms. Using fragments smaller than 20 atoms required placement using Phaser rather than the internal ACORN procedure. The resolution limits were also tested using the same 50-atom fragment, and the structure was solved using the same procedure up to a resolution of 1.15 Å. The lysozyme model could also be solved in Fragon starting from a penta-alanine fragment, the smallest fragment allowed in the CCP4i2 interface^55^.

Additional tests were set up to explore the limits of our solution under different circumstances. First, a ten-alanine (50 atoms) helix was used for molecular replacement at resolutions worse than 0.87 Å. A single helix could be placed with high accuracy to a resolution of at least 1.5 Å. Using this solution as the initial phases for the ACORN density-modification procedure using the data to 1.0 Å resulted in a map nearly identical to the one that had the helix placed using the entire resolution range. However, individual atoms were no longer resolved after density modification with data worse than a resolution of 1.15 Å. No resolution extension features were used in any runs of ACORN.

For proteinase K, ideal helices were placed using Phaser^29^. Four copies of the idealized 14-residue poly alanine helix were placed in space groups 89–96. SHELXE was run by using the merged intensities using the MTZ2HKL utility. Surprisingly, using the unmerged intensities directly from XSCALE did not result in a successful trace. Of the attempts of SHELXE from all the space groups, only space group 96 (*P*4_3_2_1_2) resulted in a well-traced structure with clear side chain density. From here, one, two and three copies of 14-amino acid-long α-helices were placed in space group 96. This was attempted again using ten-amino acid-long helices, for which only the search for four copies resulted in a convincing solution. SHELXE was run with or without the ‘-q’ option to first search for helix shapes during the chain tracing. The first solution from four helices in space group 96 used the SHELXE command line ‘shelxe 1.pda –s0.4 –a30.’ Here, the default ten rounds of density modification are followed by standard chain tracing. This is repeated 30 times. Among these trials, only the four placed 14-amino acid-long helices gave an obvious solution after chain tracing. However, the solution in space group 96 with three helices placed was also able to give a similar solution upon adding the ‘–o’ option to trim away the low CC amino acids, ‘-q’ to search for helical shapes and increasing the chain tracing rounds to 50 or 100.

For both lysozyme and proteinase K after the last round of density modification, the protein was built automatically by Buccaneer^20^. For lysozyme, the entire protein was built into the map produced by ACORN^19^ except for two terminal residues that were not resolved upon inspection of the map in Coot^56^. For proteinase K, the traced backbone from SHELXE was used as a starting fragment for Buccaneer. In both cases, electron scattering factors were used for the maps. The built structures were refined in REFMAC^31^ using electron scattering factors calculated by the Mott–Bethe formula. Initial refinements used isotropic atomic displacement (*B*) factors for individual atoms, and water molecules were added automatically. Refinement was always followed by manual curation of the model using Coot. For lysozyme, NO_3_^−^ ions were found in multiple locations that were not adequately modeled by single water molecules. For proteinase K, two I3C molecules with low occupancies were identified after the structure was entirely built and placed manually in Coot using the fragment code I3C. Hydrogen atoms were added to the lysozyme model in their riding positions for the final rounds of refinement. Once the model was completely built, the models were refined again using anisotropic atomic displacement parameters for all but the hydrogen atoms.

### Simulating the DQE for a single pixel during 250 frames

The simulated DQE is calculated as the ratio of the number of counted electrons, *N*_out_, and the number of incoming electrons, *N*_in_. It is assumed that the detector does not overcount, that is, *N*_out_ ≤ *N*_in_ and DQE ≤ 1. However, if two or more electrons arrive during the same frame, undercounting occurs because the pixel records at most one electron per frame. Over a 250-frame interval, *N*_out_ will be equal to the number of frames that recorded at least one electron. By choosing the frame randomly from a uniform distribution in the range (1, 250) for each of the *N*_in_ incoming reflections, the DQE can be simulated. To establish the DQE curve, the 250-frame exposure was simulated 1,000 times for each value of *N*_in_. This code is available in the Supplementary Code.

### Comparisons of MicroED and X-ray structures

The observed amplitudes for triclinic lysozyme determined here and by X-ray diffraction to similar resolutions were compared (Supplementary Fig. 4). Observed structure factor amplitudes were compared against those from PDB 4LZT^30^. The calculated structure factor amplitudes were calculated using the proper scattering factor libraries using SFALL^27^. Intensities from the deposited X-ray structures were converted to amplitudes using CTRUNCATE^27^. Cross-correlation scatterplots with best fit lines and the correlation coefficients between datasets were calculated using Microsoft Excel.

### Statistics and reproducibility

Twenty crystals of lysozyme and five proteinase K crystals were identified and milled. Of these, 18 lysozyme crystals and four proteinase crystals survived milling and transfer between the FIB–SEM and TEM. All lamellae diffracted, and data were collected. Of those collected, two lysozyme datasets and two proteinase datasets were discarded during the merging stage for deteriorating the quality of the merge. For simulation of the camera DQE, simulations were repeated 256 times and averaged. The standard deviation is given as a band in the plot of Fig. 1d.

### Figure preparation

Figures were prepared using ChimeraX and PyMOL and then arranged in PowerPoint. Tables were arranged in Excel. Images were cropped around areas of interest, and brightness and contrast were adjusted in Fiji^57^.

### Software availability

Software tools that convert summed or unsummed MRC stacks to Super Marty View or TIFF format are available at https://cryoem.ucla.edu/MicroED.

### Reporting Summary

Further information on research design is available in the Nature Research Reporting Summary linked to this article.

## Online content

Any methods, additional references, Nature Research reporting summaries, source data, extended data, supplementary information, acknowledgements, peer review information; details of author contributions and competing interests; and statements of data and code availability are available at 10.1038/s41592-022-01485-4.

## Supplementary information

Supplementary Information Supplementary Figs. 1–6, Tables 1–3 and Code

Reporting Summary

Supplementary Video 1 A rotation movie showing the normalized structure factor maps after density modification over the final structure of lysozyme at a resolution of 0.87 Å. The movie was sliced to a width of 8 Å.

Supplementary Video 2 A rotation movie showing the normalized structure factor maps after density modification over the final structure showing a region rich with hydrophobic packing interactions. A tryptophan–isoleucine–tryptophan sandwich is shown with several other tryptophan residues flanking this region. The movie was sliced to a width of 8 Å.

Supplementary Software Software to simulate the DQE in a diffraction spot in counting mode

Supplementary Tables Merging statistics for triclinic lysozyme, MicroED crystallographic table of proteinase K, Merging statistics for proteinase K

## Data Availability

Model coordinates and structure factors for triclinic lysozyme and proteinase K structures have been deposited to the PDB under accession codes 7SKW and 7SKX, respectively. Maps for lysozyme and proteinase K have been deposited to the EMD under accession codes EMD 25184 and EMD 25185, respectively. Source data are provided with this paper.

## Source data

Source Data Fig. 1 Data used to generate the inset in Fig. 1c and Fig. 1d.
