# Supplementary material for: Ab initio phasing macromolecular structures using electron-counted MicroED data
Source: Nat Methods. 2022 May 30;19(6):724–9. doi: 10.1038/s41592-022-01485-4 (PMC9184278; doi:10.1038/s41592-022-01485-4)
Supplement: Supplementary file 1 — Supplementary Figs. 1–6, Tables 1–3 and Code [file 41592_2022_1485_MOESM1_ESM.pdf]

nature portfolio

https://doi.org/10.1038/s41592-022-01485-4

Supplementary information

Ab initio phasing macromolecular structures using electron-counted MicroED data

In the format provided by the authors and unedited

Supplementary information

Supplementary Figure 1. Histogram of a MicroED dataset collected on the Falcon 4. Logarithmic histograms of intensities from both proteinase K datasets. (Left) low-count region showing resolved individual counts. (Right) Higher count region showing the distribution of counts below the indicated theoretical DQE threshold. No pixel counts fell below a DQE value of 0.6 for these higher dose datasets.

Supplementary Figure 2. Electron counted MicroED data from milled lamellae of proteinase K. (A) A typical proteinase K microcrystal imaged using the focused ion beam. **(B)** A thin, milled lamella from (A) identified in the TEM. **(C)** MicroED data collected in counting mode on a Falcon 4 direct electron detector from a proteinase lamella.

Supplementary Figure 3. Plots of integration statistics for lysozyme.

Supplementary Figure 4. Comparison of MicroED and X-ray structure factors. Plots comparing the observed and calculated structure factor amplitudes for triclinic lysozyme determined by MicroED and X-ray.

Supplementary Figure 5. Plots of integration statistics for proteinase K.

Supplementary Figure 6. ***Ab initio* structure of Proteinase K at 1.5 Å resolution. (Top)** The progression of the models during the phasing process. Four, 14 alanine fragments with idealized geometry were chosen as the starting fragments. The fragments were placed using molecular replacement. From this initial placement, repeated density modification and chain tracing were conducted. The chain traced model was used as an input for automatic building, and the entire protein structure was completed automatically. **(Bottom)** maps at the intermediate stages of the phasing process. $2mF_o$-DF_c maps are in white and contoured at the 1.0 σ level, whereas the mF_o-DF_c maps are contoured at the +/- 3.0 σ levels and presented in green and red, respectively.

Supplementary Table 1. Merging statistics for triclinic lysozyme

Supplementary Table 2. MicroED crystallographic table of of proteinase K

Supplementary Table 3. Merging statistics for proteinase K

Supplementary Movie 1. A rotation movie showing the normalized structure factor maps after density modification over the final structure of lysozyme at 0.87 Å resolution. The movie is sliced to a width of 8 Å.

Supplementary Movie 2. A rotation movie showing the normalized structure factor maps after density modification over the final structure showing a region rich with hydrophobic packing interactions. Tryptophan, isoleucine, tryptophan sandwich are shown with several other tryptophan residues flanking this region. The movie is sliced to a width of 8 Å.

Supplementary Python Code. Code used to simulate the DQE for the Falcon 4 direct electron detector in a diffraction spot.

```
#! /usr/bin/env python3
##Python code to simulate DQE for counting mode in a diffraction spot
import numpy as np
from math import sqrt
from sys import stderr
# Number of trials.
M = 256
# Maximum number of electrons per second.
N_max = 16384
# Framerate of the detector in Hz.
f = 250
# Low limits inclusive, high limits exclusive for both randint() and
# range().
trials = np.zeros((N_max, 2))
print("Trial ", end='', file=stderr)
for i in range(M):
    if i % 10 == 0:
        print(f"{i}... ", end='', file=stderr)
        stderr.flush()
    for N_in in range(1, N_max):
        N_out = np.unique(np.random.randint(0, high=f, size=N_in)).size
        dqe = N_out / N_in

        trials[N_in, 0] += dqe
        trials[N_in, 1] += dqe**2
print("", file=stderr)
```

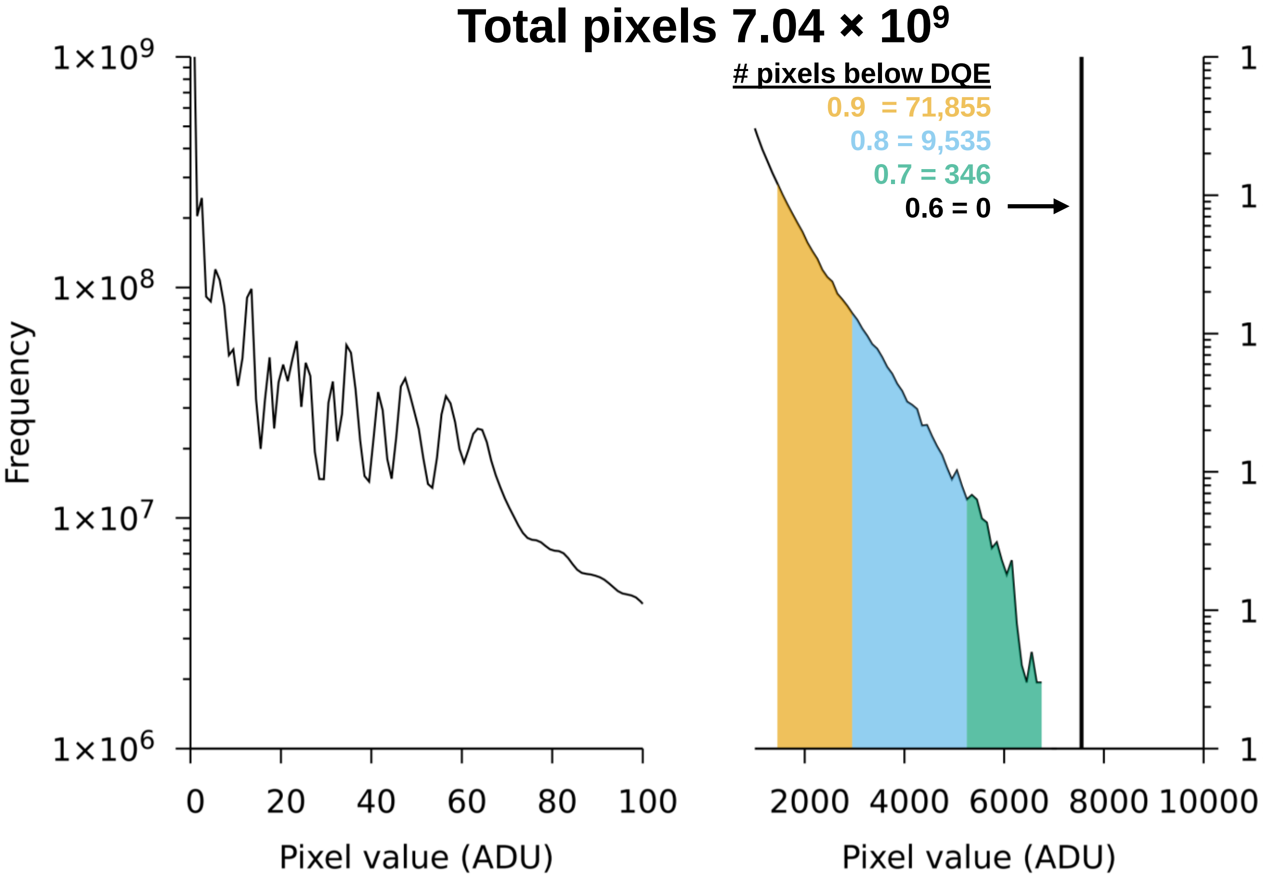

Supplementary Figure 1. Histogram of a MicroED dataset collected on the Falcon 4. Logarithmic histograms of intensities from both proteinase K datasets. (Left) low-count region showing resolved individual counts. (Right) Higher count region showing the distribution of counts below the indicated theoretical DQE threshold. No pixel counts fell below a DQE value of 0.6 for these higher dose datasets.

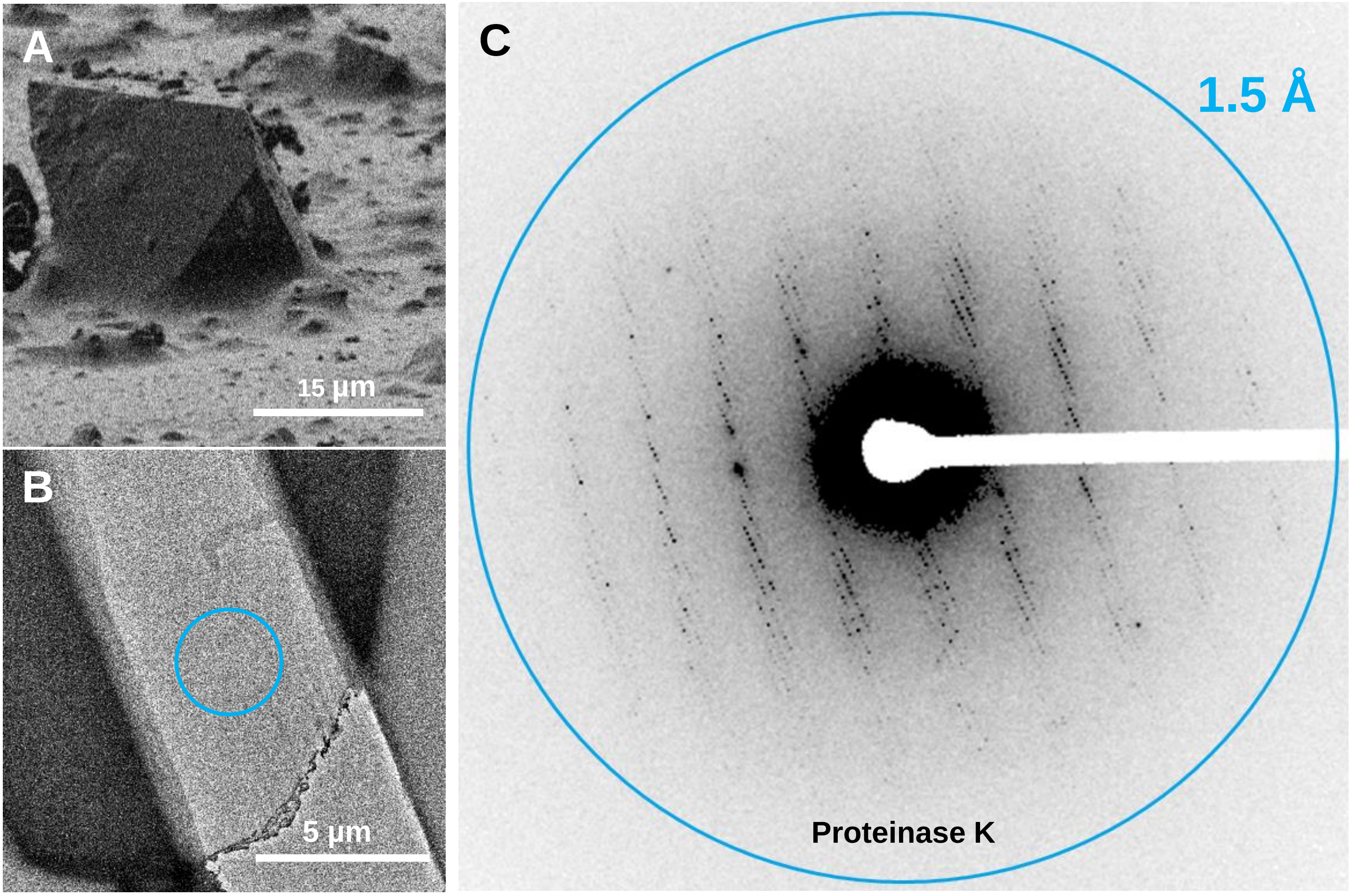

Supplementary Figure 2. Electron counted MicroED data from milled lamellae of proteinase K. (A) A typical proteinase K microcrystal imaged using the focused ion beam. **(B)** A thin, milled lamella from (A) identified in the TEM. **(C)** MicroED data collected in counting mode on a Falcon 4 direct electron detector from a proteinase lamella.

Plots of completeness, CC1/2, ⟨I/σ⟩, and R-pim for lysozyme.

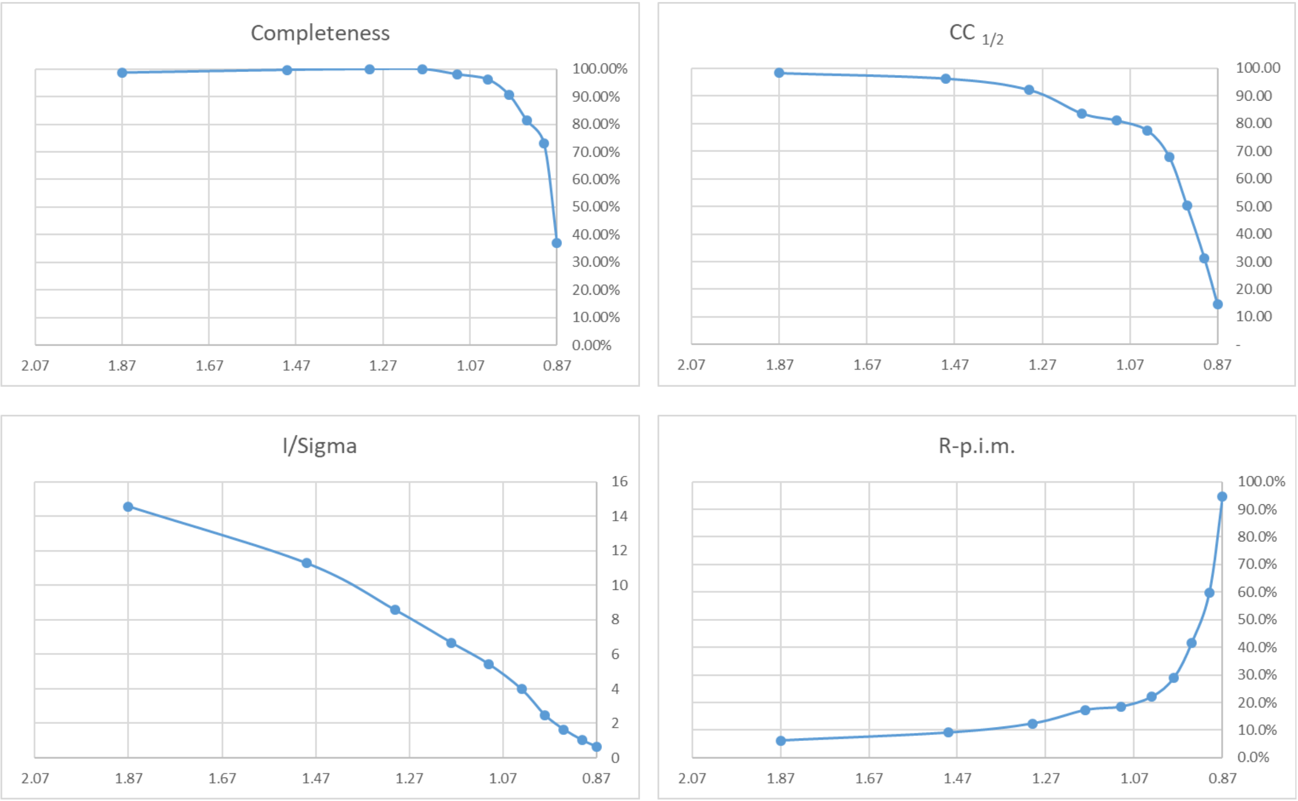

Supplementary Figure 3. Plots of integration statistics for lysozyme.

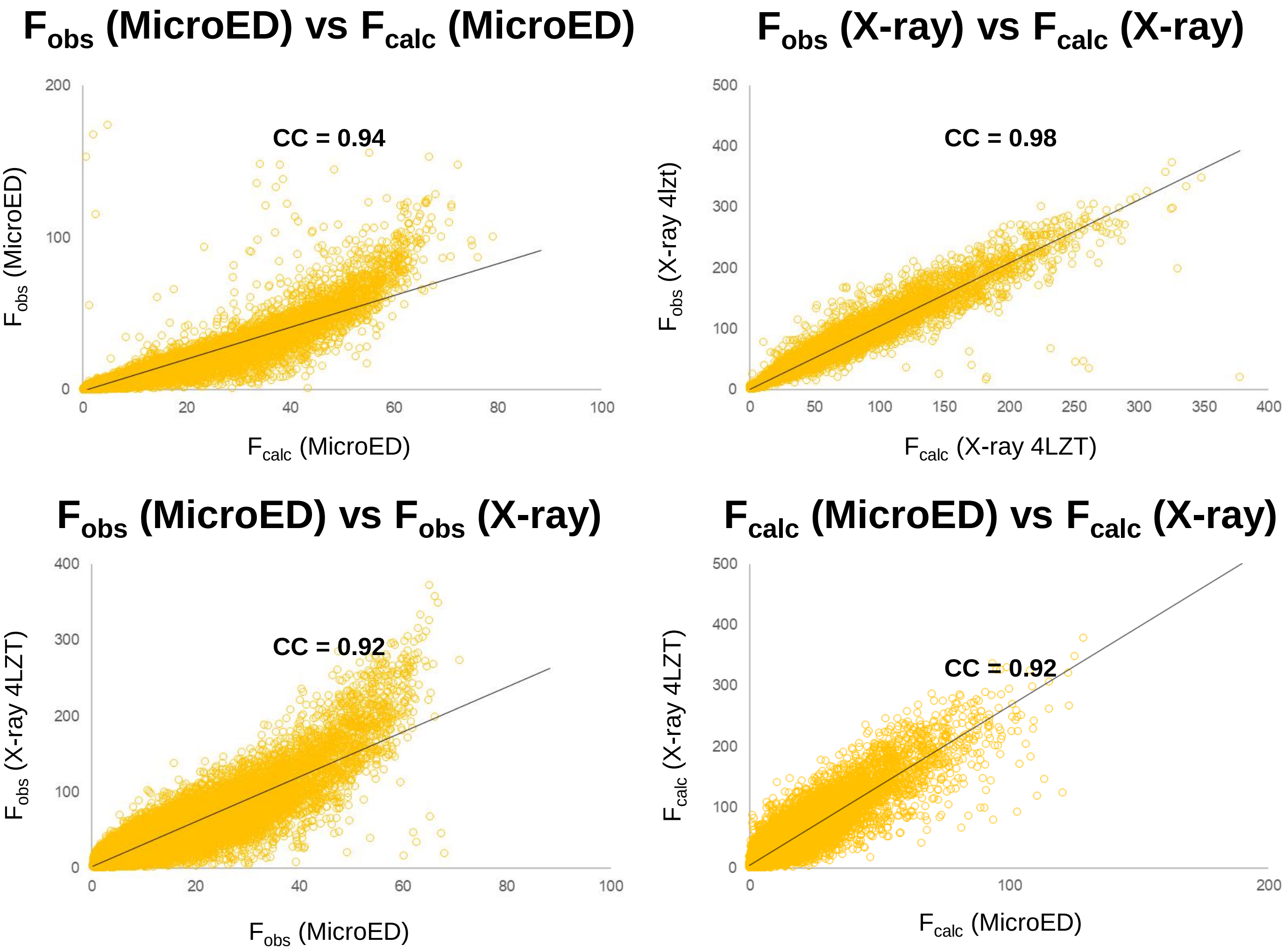

Supplementary Figure 4. Comparison of MicroED and X-ray structure factors. Plots comparing the observed and calculated structure factor amplitudes for triclinic lysozyme determined by MicroED and X-ray.

Plots of completeness, CC1/2, ⟨I/σ⟩, and R-pim for proteinase

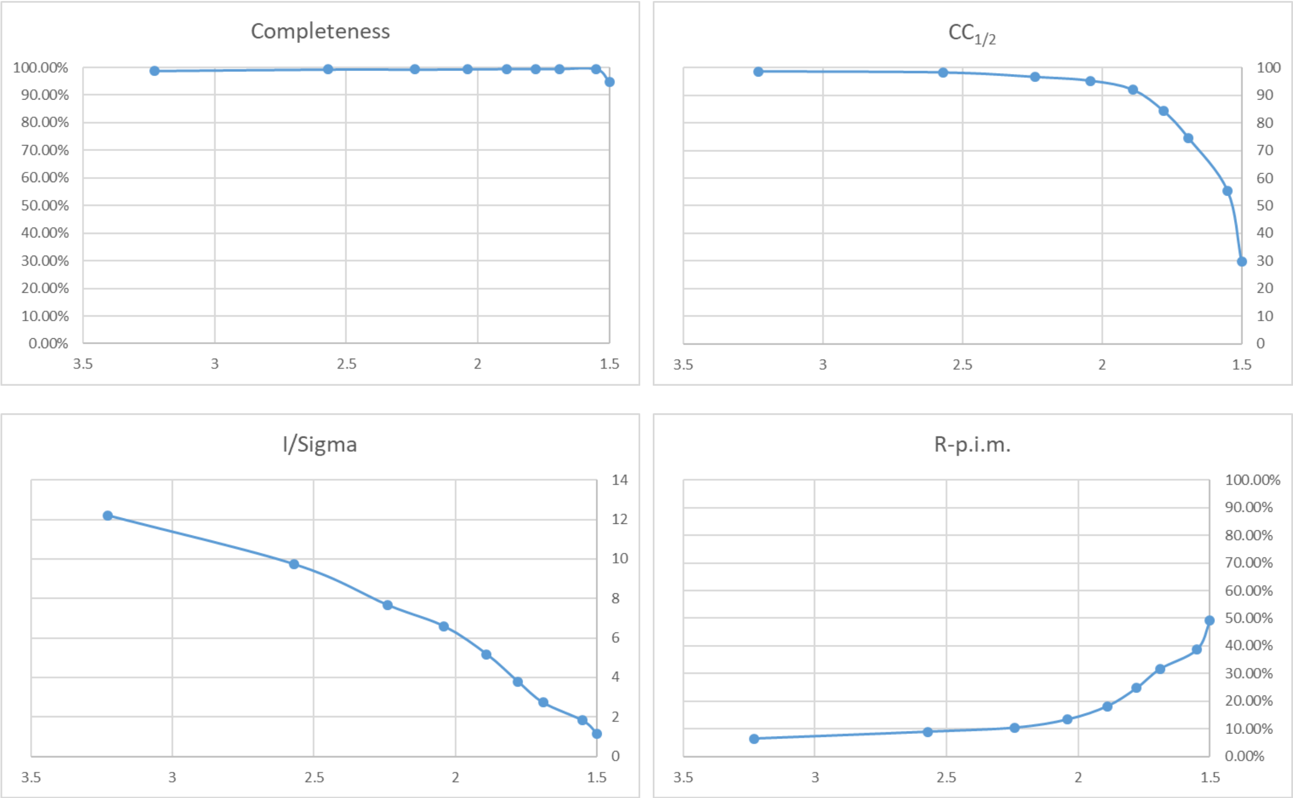

Supplementary Figure 5. Plots of integration statistics for Proteinase K.

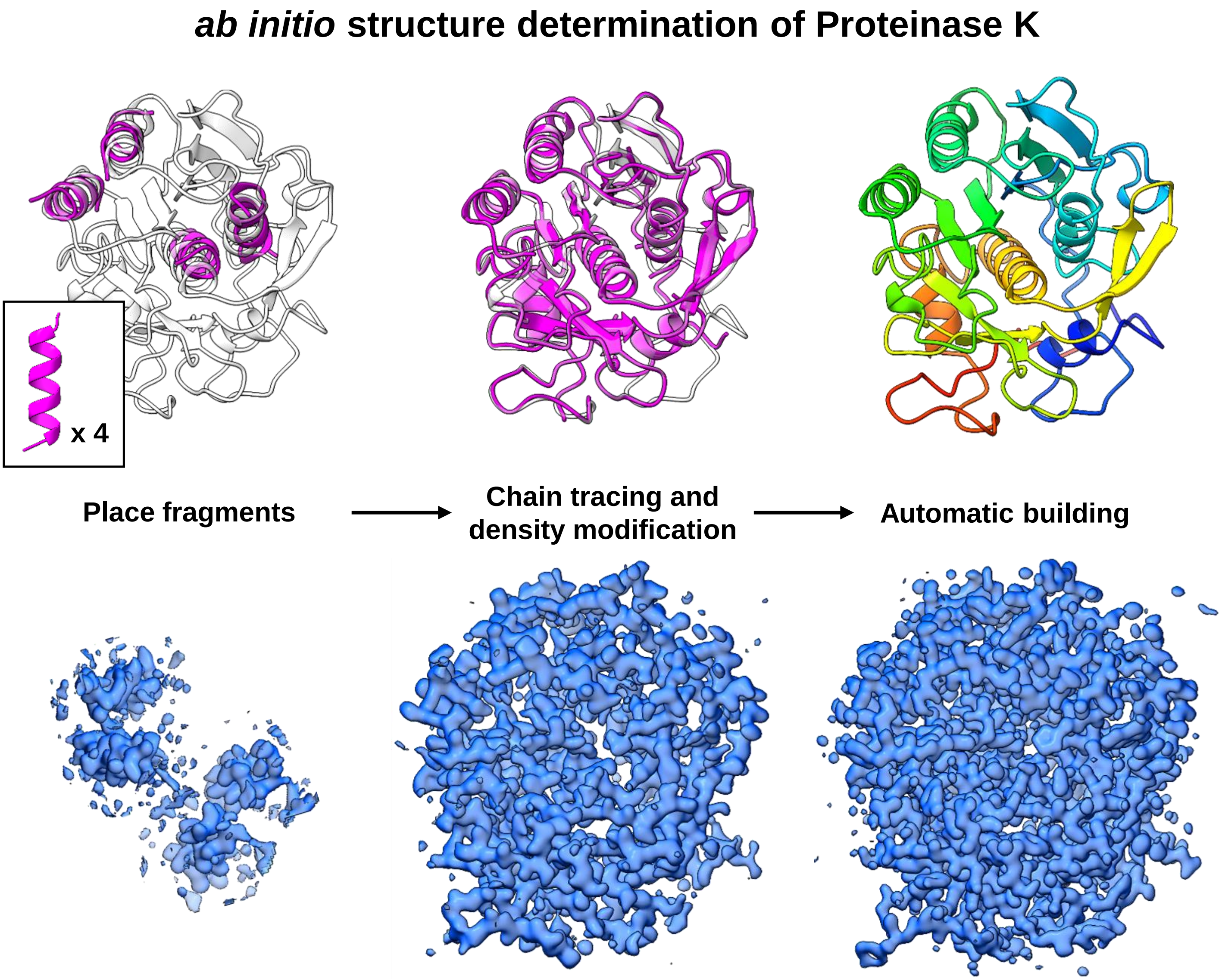

Supplementary Figure 6. Ab initio structure of Proteinase K at 1.5 Å resolution. (Top) The progression of the models during the phasing process. Four, 14 alanine fragments with idealized geometry were chosen as the starting fragments. The fragments were placed using molecular replacement. From this initial placement, repeated density modification and chain tracing were conducted. The chain traced model was used as an input for automatic building, and the entire protein structure was completed automatically. (Bottom) maps at the intermediate stages of the phasing process. 2mFo-DFc maps are in white and contoured at the 1.0 σ level, whereas the mFo-DFc maps are contoured at the +/- 3.0 σ levels and presented in green and red, respectively.
