# Supplementary material for: Ab initio phasing macromolecular structures using electron-counted MicroED data
Source: Nat Methods. 2022 May 30;19(6):724–9. doi: 10.1038/s41592-022-01485-4 (PMC9184278; doi:10.1038/s41592-022-01485-4)
Supplement: Supplementary file 2 — Reporting Summary [file 41592_2022_1485_MOESM2_ESM.pdf]

nature portfolio

Corresponding author(s): Tamir Gonen

Last updated by author(s): Mar 23, 2022

Reporting Summary

Nature Portfolio wishes to improve the reproducibility of the work that we publish. This form provides structure for consistency and transparency in reporting. For further information on Nature Portfolio policies, see our Editorial Policies and the Editorial Policy Checklist.

Statistics

For all statistical analyses, confirm that the following items are present in the figure legend, table legend, main text, or Methods section.

n/a	Confirmed	
☐	☒	The exact sample size (*n*) for each experimental group/condition, given as a discrete number and unit of measurement
☐	☒	A statement on whether measurements were taken from distinct samples or whether the same sample was measured repeatedly
☐	☒	The statistical test(s) used AND whether they are one- or two-sided<br>*Only common tests should be described solely by name; describe more complex techniques in the Methods section.*
☐	☒	A description of all covariates tested
☐	☒	A description of any assumptions or corrections, such as tests of normality and adjustment for multiple comparisons
☐	☒	A full description of the statistical parameters including central tendency (e.g. means) or other basic estimates (e.g. regression coefficient) AND variation (e.g. standard deviation) or associated estimates of uncertainty (e.g. confidence intervals)
☒	☐	For null hypothesis testing, the test statistic (e.g. F, t, r) with confidence intervals, effect sizes, degrees of freedom and P value noted<br>*Give P values as exact values whenever suitable.*
☒	☐	For Bayesian analysis, information on the choice of priors and Markov chain Monte Carlo settings
☒	☐	For hierarchical and complex designs, identification of the appropriate level for tests and full reporting of outcomes
☒	☐	Estimates of effect sizes (e.g. Cohen's *d*, Pearson's *r*), indicating how they were calculated

Our web collection on statistics for biologists contains articles on many of the points above.

Software and code

Policy information about availability of computer code

Data collection	FIB/SEM data were collected using the user interface software, microscope control v13.4.2 of a ThermoFisher Aquilos system. MicroED data were collected manually via the camera user interface software, VELOX v3.0.0 on a ThermoFisher Krios 3Gi transmission electron microscope.
Data analysis	XDS version Feb 5 2021 BUILT=20210323<br>PHASER 2.8.3<br>ACORN v2<br>CCP4 7.1.016<br>REFMAC v5.8.0049<br>MicroED tools v0.0.3 build 20201231<br>SHELXE v2019/1<br>BUCCANEER from CCP4 build 7.1.016

For manuscripts utilizing custom algorithms or software that are central to the research but not yet described in published literature, software must be made available to editors and reviewers. We strongly encourage code deposition in a community repository (e.g. GitHub). See the Nature Portfolio guidelines for submitting code & software for further information.

Data

Policy information about availability of data

All manuscripts must include a data availability statement. This statement should provide the following information, where applicable:

- Accession codes, unique identifiers, or web links for publicly available datasets
- A description of any restrictions on data availability
- For clinical datasets or third party data, please ensure that the statement adheres to our policy

Model coordinates and structure factors for triclinic lysozyme and proteinase K structures have been deposited to the PDB with accession codes 7SKW and 7SKX, respectively. Maps for lysozyme and proteinase have been deposited to the EMDB with accession codes EMD-25184 and EMD-25185, respectively. Data for individual plots and tables presented are included as Extended Data with this manuscript. Other data are available from the authors upon request.

Field-specific reporting

Please select the one below that is the best fit for your research. If you are not sure, read the appropriate sections before making your selection.

☒ Life sciences ☐ Behavioural & social sciences ☐ Ecological, evolutionary & environmental sciences

For a reference copy of the document with all sections, see nature.com/documents/nr-reporting-summary-flat.pdf

Life sciences study design

All studies must disclose on these points even when the disclosure is negative.

Sample size	20 lysozyme (n=20) and 5 proteinase K (n=5) crystals were selected for this study. Samples were selected based on their random availability upon the EM grids. Successful ab initio phasing and structure solution dictated that no further samples were required for this study. No sample size calculations were performed prior to data collection.
Data exclusions	Crystal datasets were discarded if they scaled with a correlation coefficient below 95% with other datasets. Two lysozyme datasets and two proteinase K datasets were discarded.
Replication	Small variations may arise in crystal growth and crystals deteriorate during data collection due to radiation damage. Therefor, each crystal was measured only once. Repetition of n=20 and n=5 for the two samples served as the only form of replication conducted.
Randomization	Test reflections equally 5% of the total reflections in each scaled dataset were randomly generated for validation. Calculation of random half datasets was conducted using standard crystallographic software listed herein without user intervention. Randomization of crystal samples is not relevant to structure determination studies.
Blinding	Investigators were not blinded to group allocation as no groups were allocated in this study.

Reporting for specific materials, systems and methods

We require information from authors about some types of materials, experimental systems and methods used in many studies. Here, indicate whether each material, system or method listed is relevant to your study. If you are not sure if a list item applies to your research, read the appropriate section before selecting a response.

Materials & experimental systems

n/a	Involved in the study	
☒	☐	Antibodies
☒	☐	Eukaryotic cell lines
☒	☐	Palaeontology and archaeology
☒	☐	Animals and other organisms
☒	☐	Human research participants
☒	☐	Clinical data
☒	☐	Dual use research of concern

Methods

n/a	Involved in the study	
☒	☐	ChIP-seq
☒	☐	Flow cytometry
☒	☐	MRI-based neuroimaging
